# Myocarditis Related to COVID-19 and SARS-CoV-2 Vaccination

**DOI:** 10.3390/jcm11236999

**Published:** 2022-11-26

**Authors:** Ana I. Molina-Ramos, Elisabeth Gómez-Moyano, Jorge Rodríguez-Capitán, María Angullo-Gómez, Patricia Gallardo-Jiménez, Iván Pérez de Pedro, Lucía Valiente de Santis, Beatriz Pérez-Villardón, Isabel Piñero-Uribe, Javier Mora-Robles, Víctor Manuel Becerra-Muñoz, Manuel Jiménez-Navarro

**Affiliations:** 1 Centro de Investigación en Red de Enfermedades Cardiovasculares (CIBERCV), Cardiology Department, Hospital Universitario Virgen de la Victoria, 29010 Málaga, Spain; 2 IBIMA-Plataforma BIONAND, Universidad de Málaga, 29590 Málaga, Spain; 3 Dermatology Department, Hospital Regional Universitario de Málaga, 29010 Málaga, Spain; 4 Internal Medicine Department, Hospital Regional Universitario de Málaga, 29010 Málaga, Spain; 5 Infectious Diseases Department, Hospital Regional Universitario de Málaga, 29010 Málaga, Spain; 6 Cardiology Department, Hospital Regional Universitario de Málaga, 29010 Málaga, Spain

**Keywords:** COVID-19, myocarditis, mRNA vaccines

## Abstract

The coronavirus disease of 2019 (COVID-19) has been a cause of significant morbidity and mortality worldwide. Among the short- and long-term consequences of COVID-19, myocarditis is a disease to be taken into consideration. Myocarditis, in general, is related to a poor prognosis. However, the epidemiology and prognosis of myocarditis related to COVID-19 are currently unknown. While vaccination against COVID-19 is of great benefit at a public health level, the risk of myocarditis should be considered in the context of the global benefits of vaccination. In this narrative review, we will summarize the etiopathogenic bases, the epidemiology, the clinical manifestations, the course, diagnosis, prognosis, and the treatment of myocarditis related to SARS-CoV-2, as well as myocarditis secondary to mRNA vaccines.

## 1. Introduction

Myocarditis is an inflammatory disease of the cardiac muscle and, in the most cases, it is secondary to viral infections. Currently, SARS-CoV-2 is emerging as an additional causative agent of myocarditis and its pathogenesis is still unknown. The main hypothesis postulates that, in certain individuals, the immune response against the glycoprotein spike could confer a risk of immune-mediated damage, because of the molecular mimicry between SARS-CoV-2 proteins and the myocardium [1]. The alternative hypothesis posits that the myocardial damage could be due to a nonspecific inflammatory response secondary to SARS-CoV-2 vaccination with a direct role by the ACE-2 receptor [1,2].

Previous research has shown that a high value of serum troponin in COVID-19 patients is associated with an increase in mortality. However, as is well known, an isolated high value of serum troponin is not suitable for the diagnosis of acute myocarditis, and it is necessary to use the presence of other analytical findings, such as lymphopenia, elevated D-dimer, ferritin, and/or C-reactive protein together with high clinical suspicion [3].

The incidence and prognosis of this new entity is currently unknown and new multicenter registries will be necessary to confirm it. The early detection of residual myocardial damage in COVID-19 survivors is one of the main objectives in order to identify patients at risk of developing cardiovascular complications [4]. While vaccination against COVID-19 shows great benefits at the public health level, the risk of possible myocarditis should be considered over the benefits of global vaccination [5].

**Figure 1:**
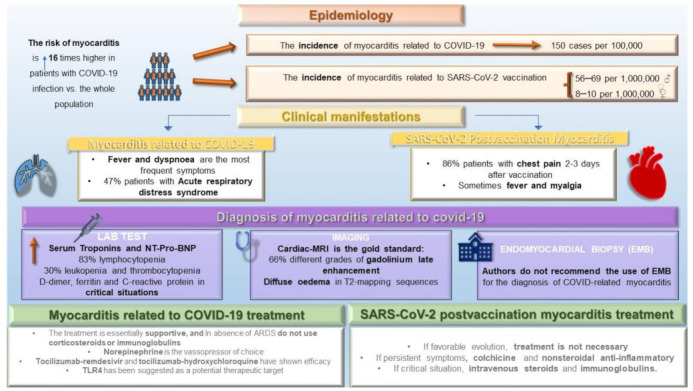
Graphical description of the epidemiology, clinical manifestations, diagnosis, and treatment of myocarditis related to COVID-19 and SARS-CoV-2 vaccination. ARDS = acute respiratory distress syndrome.

Thus, in this narrative review, we will review the etiopathogenic bases, epidemiology (incidence and risk factors), clinical manifestations/course, diagnosis, outcome, and treatment of COVID-related and SARS-CoV-2 postvaccination myocarditis. Figure 1 helps to summarize the information contained in this review. After the review of this subject, we will be able to propose working hypotheses for future research and draw conclusions that may be useful in daily clinical practice.

## 2. Materials and Methods

We searched the PubMed, Google Scholar, and Web of Science databases using the keywords: (SARS-CoV-2 OR COVID-19 OR SARS-CoV-2 vaccine OR COVID-19 vaccine) AND (myocarditis OR pericarditis). The filters used were the following: free full text, case reports, clinical study, multicenter study, observational study, humans, systematic review, and meta-analysis sorted by: most recent. Finally, a manual selection was made of the bibliographic references. The search was conducted in February 2022. A review of the titles, abstracts, and manuscripts was performed, and a total of 41 relevant articles were selected. Stress cardiomyopathy and animal studies were excluded. Table 1 shows all of the articles considered in this review, and they have been classified depending on the pandemic wave analyzed.

## 3. Etiopathogenic Bases

### 3.1. Etiopathogenic Bases of COVID-19-Related Myocarditis

The first published case-reports about COVID-19 outlined a spectrum of cardiovascular diseases, such as myocarditis, stress cardiomyopathy, myocardial infarction, and arrhythmias. The researchers concluded that the myocardial damage in COVID-19 patients seems to be secondary to the critical state rather than being due to a direct injury to the myocardium by the viral infection [3].

However, myocardial inflammation with or without direct damage of the cardiomyocytes has been proven in recent publications, which suggests that several pathophysiological mechanisms could be responsible for COVID-related myocarditis [1]. The pathogenesis has not been completely elucidated, although the two main theories predict a direct role of the ACE2 receptor [1,2] and a hyperimmune response with cytokine storm and systemic inflammation [23]. SARS-CoV-2 binds to the ACE2 receptor through its spike protein and penetrates into different types of cells, such as epithelial cells and macrophages [23]. Moreover, these receptors are also expressed in the myocardium, and mice models have showed that the SARS-CoV-2 can regulate ACE2 activity and produce myocardial inflammation [1].

Intracellular SARS-CoV-2 could affect the formation of stress granules through its accessory protein [24]. The viral replication is kept with stress granules. In addition, the antigens are presented to naïve T-lymphocytes by the antigen-presenting cells, and these antigen-presenting cells show cardiotropism by the hepatocyte growth factor (HGF) produced by the myocardium. HGF binds c-Met, an HGF receptor on T-lymphocytes [24]. CD^8+^-primed T lymphocytes migrate to cardiomyocytes and the myocardial damage is caused by cell-mediated cytotoxicity. In cytokine storm syndrome, the overactivation of T-lymphocytes entails a release of proinflammatory cytokines into the circulation, resulting in a positive feedback loop between the immune activation and the myocardial damage [24].

Hajjo et al. demonstrated a central role of interferon-gamma (INF-gamma) affecting the MAPK and JAK-STAT signaling pathways with adverse cardiac events, both myocarditis and pericarditis, based on computerized approach [17].

The expression of ACE2 can be increased by the union between SARS-CoV-2 and activated-TLR4 facilitating the entry and causing hyperinflammation. The innate immune receptor TLR4 is present on the cell surface and recognizes pathogen-associated molecular patterns (PAMPs), such as viral proteins, and triggers the production of type I interferon and proinflammatory cytokines, that participate in the immune response. ACE2 acts as the entry receptor for SARS-CoV-2, and it is only present in approximately one or two percent of lung cells. Recent studies have proposed that the spike protein presents the strongest protein–protein interaction with TLR4. Aboudounya et al. proposed that the peak glycoprotein SARS-CoV-2 binds to TLR4 and, as we mentioned above, this binding leads an overexpression of ACE2 on the cell surface. The result is the entry of SARS-CoV-2 into the cell. Therefore, TLR4 contributes significantly to the pathogenesis of SARS-CoV-2, and its overactivation causes a prolonged or excessive innate immune response and produces not only myocarditis but also multiple organ injury [2].

The COVID-related myocarditis obeys different pathophysiological mechanisms. Among them, a profound alteration of endothelial homeostasis is observed, which opens up to various therapeutic approaches. The endothelial disturbance created by viral invasion and the circulating cytokines could promote the appearance of microvascular disease manifested by oedema, microvascular inflammation, or atherothrombosis; these mechanisms are also involved in the pathophysiology of other cardiovascular diseases such as myocardial infarction, carotid artery thrombosis, cerebrovascular accidents, or pulmonary embolism [25].

Multisystem inflammatory syndrome is a hyperinflammatory response in which multiple organs can be damaged. Although some scientists have postulated the direct invasion of the virus as the most likely mechanism, others focus on the response of host inflammatory cells. Recent investigations show a maladaptive host immune response driven by overactivation of innate immune pathways along with a sudden increase in proinflammatory cytokines, deregulated thromboinflammation, thrombotic microangiopathy, and endothelial dysfunction. This inflammatory state may play a main role in the pathogenesis of cardiac damage observed in the infection by SARS-CoV-2. Other hypothetical mechanisms also contributing to this inflammatory situation include the oxygen supply–demand balance and the involvement of other comorbidities, (i.e., metabolic syndrome, hypertension, and other cardiovascular risk factors) [26].

### 3.2. Etiopathogenic Bases of Myocarditis Related to SARS-CoV-2 Vaccination

Although post-vaccination myocarditis does not have a straightforward biological explanation, the molecular mimicry between the spike protein of SARS-CoV2 and the autoantigens triggers the dysregulation of several pathways, such as: the immune responses in selected individuals, the immune response to mRNA, the activation of immunological pathways, and the deregulated expression of cytokines [26]. Therefore, the mRNA vaccine could be identified as an antigen by the human immune system in selected individuals, which leads to the activation of proinflammatory cascades and systemic reactions, thus contributing to the development of acute myocarditis [26].

## 4. Epidemiology: Frequency and Risk Factors

### 4.1. Epidemiology of COVID-19-Related Myocarditis

Myocardial damage is defined as the presence of elevated serum cardiac enzymes. It has been reported in between 19 and 28% of patients with COVID-19 infection, and in 40% of them at the beginning of the pandemic [1]. However, clinically confirmed myocarditis following the Dallas criteria (Table A1) is still quite rare in the SARS-CoV-2 infection. The incidence of myocarditis/pericarditis among 181,656 hospitalized patients, excluding patients with the diagnosis of cancer, was 0.08% (0.78 per 1000 hospitalized patients) during the follow-up period [36]. In a systematic review of 38 cases of myocarditis, 14 were older than 50 years and 24 were younger than 50 years. By sex, 24 of the 36 patients were men, compared to 12 of the 36 patients being women [1]. A 16-fold higher risk has been estimated in patients with COVID-19 infection compared to the whole population, with an incidence of approximately 150 cases per 100,000 of COVID-related myocarditis [18]. Unlike what might be expected, in a series of 51 cases published in 2020, the mean age documented in COVID-related myocarditis was 56.3 years [27]. The development of COVID-related myocarditis seems to be independent of pre-existing cardiovascular risk factors. However, it was observed that the patients with myocardial damage present more comorbidities, such as hypertension, diabetes mellitus, coronary heart disease, heart failure, kidney disease, or atrial fibrillation [36].

### 4.2. Epidemiology of Myocarditis Related to SARS-CoV-2 Vaccination

According to the Vaccine Adverse Event Notification System (VAERS) in the United States, it has been documented that the rate of postvaccination myopericarditis (Pfizer and Moderna mRNA vaccines) is 56 to 69 per million vaccinated men from 12 to 17 years of age and 8 to 10 per million vaccinated women from 12 to 17 years of age [37]. A prospective study based on this population suggests that the incidence of myopericarditis in adolescent males is higher and it could lead a more severe disease developing; however, the outcome is usually favorable [36]. The higher incidence in men could be explained by the inhibitory effect on proinflammatory T-cells of estrogen in women which reduces the immune-mediated cellular response [26].

During the eight weeks after COVID-19 vaccination in individuals of 12 to 15 years of age, 19 times more cases of myocarditis were described than expected. These observations have been also found after the second dose of the vaccine [28]. The median number of days from vaccination to the diagnosis of myocarditis was between 3.5 to 20 days [28]. According to the Advisory Committee on Immunization Practices, by 11 June 2021, around 300 million doses of the COVID-19 mRNA vaccine had been administered, and around 1226 probable cases of myocarditis/pericarditis were observed in the VAERS [26].

## 5. Clinical Manifestations

SARS-CoV-2 infection can directly or indirectly produce a series of cardiovascular complications, as well as myocarditis, including acute myocardial injury, heart failure, cardiac arrest, arrhythmia, acute myocardial infarction, Tako-tsubo cardiomyopathy, coagulation abnormalities, or even cardiogenic shock [27]. The clinical diagnosis between COVID-related myocarditis, Tako-tsubo cardiomyopathy and severe COVID-19 disease constitutes a real challenge. Dyspnea is the main symptom, and high levels of serum troponin are observed in all three clinical settings, although they differ in their pathophysiological mechanism [27].

In the systematic review carried out by Swahala et al., collected from December 2019 to June 2020, both dyspnea and fever were the most frequent symptoms, with them being found in 75% of patients [29]. Acute respiratory distress syndrome (ARDS) was found in 42% of patients [29]. In another systematic review carried out by Castiello et al., of 38 patients, approximately 45% of them had a fever at the time of presentation or a slight increase in temperature, eight patients presented with gastrointestinal symptoms, and four patients had previous or current syncope [1]. Other symptoms include chest pain, palpitations, cough, and difficulty in breathing [1,29]. Cardiogenic shock was observed in 52% of the individuals [27]. At this point, among the 51 cases of COVID-related myocarditis published up until November 2020, tachycardia (76.4%), dyspnea (74.5%), cardiogenic shock (52.9%), and fever (37.3%) were the most frequent clinical signs and symptoms reported [27]. Arrhythmias can appear in the context of COVID-related myocarditis and are able to precipitate in predisposed subjects [24]. The arrhythmic disorders can differ depending on whether they are in active or healed myocarditis.

Regarding postvaccination myocarditis, in a series of 484 cases, 86% of them had chest pain between 2 or 3 days after vaccination [6,26], which was sometimes preceded by fever and myalgia [26]. In contrast, COVID-related myocarditis can appear during the acute phase [30] or until 10 weeks after viral infection [13]. 

Myocarditis can also appear in the context of multisystem inflammatory syndrome as an excessive inflammatory response, and it could damage not only the heart but also different organs such as the lungs, kidneys, brain, skin, eyes, and the gastrointestinal system [26]. Several authors link myocarditis and post-acute or prolonged COVID sequelae due to SARS-CoV-2 infection [13]. The described symptoms cover a wide variety of cardiovascular and neurological alterations, including fatigue, palpitations, chest pain, shortness of breath, mental confusion, dysautonomia, and postural tachycardia. These findings demonstrate the strong impact of COVID infection on the human body [3].

## 6. Diagnosis

### 6.1. Diagnosis of COVID-19-Related Myocarditis

#### 6.1.1. Lab Test

The elevated serum troponins frequently observed in COVID-related myocarditis patients make the differential diagnosis between myocardial ischemia and myocarditis a real challenge, in which the clinical setting plays a main role. The finding of negative high-sensitivity serum troponin would make the diagnosis of acute myocarditis significantly less probable [24], although it can be observed in atypical forms of myocarditis, such as giant cell myocarditis or in the chronic myocarditis. The existence of elevated serum troponins associated with a negative coronary angiography should increase the suspicion of myocarditis and encourage treatment accordingly [3].

In COVID-19 patients, the value of NT-proBNP could also increase due to myocardial stress, a consequence of severe respiratory disease, and it has been described as a bad prognostic sign [23]. At this point, cardiac damage coexists more frequently with severe COVID-19 disease and multiple organ failure, while myocarditis remains anecdotal. Other serum markers often detected are lymphocytopenia (in up to 83% of the cases) or leukopenia and thrombocytopenia (both observed in approximately one-third of cases). A significant serum level of inflammatory markers (D-dimer, ferritin, and C-reactive protein) is detected in critical situations [1]. The existence of high serum troponin levels without other laboratory marker alterations suggests the presence of isolated myocarditis. In opposition, the combination with other inflammatory parameters is suggestive of multiorgan failure and a multisystemic inflammatory response.

#### 6.1.2. Electrocardiogram and Image Data

The electrocardiographic patterns are not pathognomonic, with a wide variety of arrhythmogenic settings, including sinus tachycardia, extrasystoles, ST-elevation, T-wave inversion, or no findings [24]. Transthoracic echocardiography must be the preferred imaging test performed when myocarditis is suspected [27]. Although cardiac magnetic resonance is the gold-standard imaging test for the diagnosis of acute myocarditis, it must be indicated with caution in light of its low initial prevalence [1]. In a recent systematic review, two-thirds of patients with myocarditis showed different grades of gadolinium late enhancement. In 19 of the 38 patients, diffuse oedema in T2-mapping sequences due to myocardial inflammation was observed [1]. 

#### 6.1.3. Endomyocardial Biopsy and Autopsy

While considered the gold standard for the diagnosis of myocarditis, endomyocardial biopsy (EMB) has been infrequently employed for the diagnosis of myocarditis even prior to COVID, due to a variety of justifiable reasons. Firstly, myocarditis is unfrequently found in autopsies or EMB from COVID-19 patients. Secondly, the patchy involvement makes it difficult to obtain samples. Thirdly, the diagnosis of myocarditis by EMB shows low prevalence and not well-known therapeutic implications. Due to these considerations, the authors do not recommend the systematic use of EMB for the diagnosis of myocarditis related to COVID-19 [31]. The most frequent histopathology finding is lymphocytic myocarditis. There is a correlation between high levels of serum troponin in COVID-19 patients and other pathophysiological alterations, such as diffuse infiltration of macrophages (88%), followed by right ventricular distension (19%), focal pericarditis (19%), thrombi of small vessels (19%), and endocardial thrombosis (14%). From the histological point of view, the myocarditis related to COVID-19 has been reported in between 4.5 and 14% of the cases [28].

The prevalence of myocarditis in a series of 277 autopsies of patients who died for COVID-19 infection was less than 2%. However, the presence of at least one histological finding related to COVID-19 (microthrombi, macrothrombi or inflammation, or intraluminal megakaryocytes) was up to 47% [32]. Other authors have also described nonocclusive fibrin microthrombi (without ischemic damage) and cardiac amyloidosis [19].

According to the “Association for European Cardiovascular Pathology guidelines for the diagnosis of myocarditis”, the diagnosis of myocarditis requires a patched inflammatory infiltrate and the presence of myocyte necrosis [40]. In lymphocytic myocarditis, for example, a polymerase chain reaction (PCR) in serum and myocardium is the gold-standard diagnostic test. Nasopharyngeal PCR is frequently negative. The presence of the SARS-CoV-2 virus in the biopsied myocytes is the decisive parameter to correctly assess COVID-related myocarditis and to identify the most appropriate treatment, since patients with negative viruses are more likely to benefit from immunosuppressive therapy. The presence of the SARS-CoV-2 virus in the biopsied myocytes usually responds to antiviral treatment [40].

### 6.2. Diagnosis of Myocarditis Related to SARS-CoV-2 Vaccination

Regarding SARS-CoV-2 postvaccination myocarditis, the diagnostic data are very similar, with some differentiating aspects [26]. None of the reported cases had presented thromboembolic events, thrombocytopenia, or coagulation abnormalities. These patients had also failed to present other signs and symptoms classically described, such as persistent fever, lymphadenopathy, hepatosplenomegaly, anemia, leukopenia, thrombocytopenia, hypofibrinogenemia, high levels of ferritin or multiorgan deterioration that suggests a cytokine storm, hemophagocytic or macrophagocytic lymphohistiocytosis, or activation syndrome, because of the overactivation of inflammatory cells [26].

In 484 probable cases of postvaccination myocarditis/pericarditis in young people who were reviewed and characterized by the CDC, 56.1% showed alterations in the ST-segment or T-wave in the electrocardiogram, 64% had elevated serum troponins (peaking on day 3), and 17% had abnormalities in the cardiac imaging tests [26]. In other study, the echocardiogram results were altered in 40% of cases, and only a small percentage had reduced left ventricular global systolic function [7].

Patients presented an immunization pattern, with positive antibodies against the spike protein and negative antibodies against the nucleocapsid, when they had not had a previous infection. Except in one case, the levels of neutralizing IgM and IgG antibodies against the SARS-CoV-2 spike were very similar in patients with post-vaccination myocarditis as opposed to vaccinated people without myocarditis [6], which presents an argument against a hyperimmune response. The patients with myocarditis also showed high levels of IL-1 receptor antagonist (interleukin 1), IL-5, and IL-16 but no proinflammatory cytokines, for example, IL-6, tumor necrosis factor, IL-1B, IL-2 or interferon-γ [26]. For the diagnosis of COVID-related myocarditis or postvaccination myocarditis, the study should be completed with a PCR test and viral serology for other causes, such as hepatitis, Epstein–Barr virus, cytomegalovirus, parvovirus, mycoplasma, HIV, influenza A/B, respiratory syncytial virus, rhinovirus, enterovirus (Coxsackie A, Coxsackie B), and adenovirus. It is also advisable to analyze the presence of autoimmune diseases with antinuclear antibodies and rheumatoid factor. In postvaccination myocarditis, these data were negative, without evidence of predilection for people with pre-existing autoimmune diseases. In these cases, the presence of leukocytosis, eosinophilia, anemia, thrombocytopenia, or elevated transaminases has not been demonstrated [26].

Surprisingly, high levels of D-dimer were found in two patients in absence of pulmonary embolism or venous thrombosis [8,14]. Moreover, one case report showed that the panel of 121 genetic variants potentially associated with cardiomyopathy was negative [9]. This fact is against the hypothesis of a pre-existing genetic predisposition to cardiomyopathy.

Finally, in the autopsy of a 22-year-old man, who presented with chest pain 5 days after his first BNT162b2 mRNA vaccine dose, the cardiac histological examination showed isolated atrial myocarditis, predominantly of the neutrophils and histiocytes. Immunohistochemical staining of C4d revealed scattered unicellular necrosis of cardiomyocytes that was not accompanied by inflammatory phenomena. Extensive necrosis of the contraction band was evidenced in the atria and in the ventricular myocardium. The presence of microthrombosis or cardiac infection was not demonstrated. It was determined that the main cause of death was acute myocarditis, and it was related to the BNT162b2 vaccine [10].

In Table 2 the main articles about the diagnosis of myocarditis and pericarditis in patients related to COVID-19 and SARS-CoV-2 vaccination have been summarized.

## 7. Treatment

### 7.1. Basis for Treatment for COVID-Related Myocarditis

Although COVID-related myocarditis treatment is essentially supportive, antiviral therapy and immunomodulators cannot be dismissed. Patients with new-onset heart failure should receive a pharmacological combination of vasopressors, diuretics, inotropes, vasodilators, and fluids as part of their treatment [23].

Taking into consideration the latest COVID-19 treatment guidelines, in critically ill patients requiring vasopressor support, norepinephrine is the drug of choice. When the target mean arterial pressure is not reached, vasopressin may be added. Because of the increased risk of arrhythmias and the mortality associated with dopamine, its use is not recommended. In patients with cardiogenic shock secondary to COVID-19 infection, the inotropic of choice is dobutamine, with milrinone as a second line in case of refractoriness [41].

In contrast, in the absence of ARDS the use of corticosteroids and intravenous immunoglobulins is discouraged [41]. However, there is room for the use of corticosteroids in those patients with lymphocytic myocarditis. In a systematic review carried out by Sawalha et al., 5 out of the 7 patients who were treated with corticosteroids recovered [29]. This is superimposable to those cases with COVID-19 infection, in whom systemic corticosteroids are recommended in cases with ARDS on mechanical ventilation, where dexamethasone has demonstrated efficacy [41]. However, the use of corticosteroids can decrease viral clearance [29]. Nevertheless, the use of corticosteroids remains under discussion. In the systematic review conducted by Sawalha et al., both corticosteroids (in 50% of the cases) and other second-line drugs such as tocilizumab (14%), immunoglobulins (21%), and interferon (14%) were used for treatment [29].

A published case-report of acute fulminant myocarditis in a patient with COVID-19 treated during 4 days with immunoglobulin (as a regulator of immunological status in a dose of 20 g/day) and methylprednisolone (as inflammation suppressor in a dose of 200 mg/day), yielded crucial information on the use of immunomodulators in patients with COVID-19 infection [11]. In children, the use of immunoglobulins as part of the treatment for myocarditis in systemic inflammatory syndrome has shown efficacy [33]. Before the pandemic, in 2019, a meta-analysis which compared IVIG against corticosteroids in the context of acute myocarditis demonstrated that those patients who received IGIV therapy showed an inferior mortality rate and a higher recovery of left ventricular systolic function [35].

Dabbagh et al. outlined that cytotoxic pathways, which are immune-mediated, can result in cardiac tamponade [12]. The GRECCO-19 trial demonstrated that colchicine, one of the well-known therapeutic agents in the treatment of pericarditis, improved the time to clinical worsening in COVID-19 patients [38]. 

Other researchers are studying the role of heparin as part of COVID-19 treatment, not merely due to its anticoagulant effects but also for its anti-inflammatory effects, inhibiting HMGB-lipopolysaccharide. Its use would have an effect on the damage of the cardiac microcirculation and on the prothrombotic state [15]. Heparin may be a defining factor in the evolution and prognosis of the disease in patients with COVID-related myocarditis. [21]. The fact that the binding of heparin to spike glycoprotein inhibits SARS-CoV2 infection has led to the proposal of heparin as a drug with antiviral activity [22].

### 7.2. Immunosuppressive Therapy for COVID-19-Related Myocraditis

Although no clinical trial has been specifically designed for this purpose at this time, recent studies aim to study which immunosuppressive therapy could provide benefits in the hyperinflammatory phase of myocarditis secondary to COVID-19 [1].

Tocilizumab, an IL-6 receptor antagonist drug used in cytokine release syndrome, has been used in a single-center case series involving 15 patients, of whom 11 showed clinical improvement or stabilization after the use of tocilizumab; however, the number of patients included in the study was low and the results were reported only one week after initiation of treatment. Four patients of the reported cases had high IL-6 levels, half of whom received tocilizumab with mixed results [20]. For the time being, several lines of research are underway to study the efficacy of IL-6 antagonist drugs in COVID-19 infection. Efficacy has been observed in the combinations of tocilizumab-remdesivir and tocilizumab-hydroxychloroquine when used as part of treatment for patients with COVID-19; however, the greater need for ICU treatement or mechanical ventilation in the TCZ-RMV arm contributed to the occurrence of cardiac and thrombotic events, including a rate of 15.4% in patient with myocarditis [39].

In a series of 15 patients with post-COVID chronic myoendocarditis, researchers demonstrated the presence of persistent SARS-CoV2 in the myocardium in combination with a hyperimmune response. Endocarditis can manifest as infectious or nonbacterial thromboendocarditis. The authors propose to study the possibility of using corticosteroids and anticoagulants in the treatment of post-COVID myoendocarditis [21].

Nonsteroidal anti-inflammatory drugs are not recommended because they can worsen the clinical course of COVID-19 patients and exacerbate kidney and myocardial damage. It is recommended for inotropic negative drugs to be avoided in patients with sinus tachycardia [24].

Finally, after studying the role of TLR4 antagonists in sepsis and other antiviral contexts, TLR4 has been suggested as a potential therapeutic target in COVID-19. As a consequence, there may be room for TLR4 antagonists in COVID-19 management [2].

### 7.3. Treatment of Myocarditis Related to SARS-CoV-2 Vaccination

Regarding postvaccination myocarditis, it has been proposed that if the interval between vaccination doses is increased, the risk of inflammatory adverse reactions would be reduced [17]. The use of systemic corticosteroids has been proposed as the preferred treatment based on the biology of the inflammatory process [17]. Among patients with a favorable evolution of symptoms with no ventricular dysfunction or cardiac biomarkers anomalies. the onset of treatment can be delayed. On the other hand, for patients with persistent symptoms but with no life-threatening situations present (left ventricular dysfunction, hemodynamic instability, arrhythmia, etc.), nonsteroidal anti-inflammatory drugs and colchicine can be contemplated. In patients with these aforementioned life-threatening cardiac situations, intravenous steroids and immunoglobulin can be added to other circulatory support measures. If left ventricular systolic dysfunction develops, additional therapy should be assessed, always directed by guidelines, which includes β-blockers and angiotensin-converting enzyme inhibitors [26].

## 8. Evolution

The clinical course of COVID-related myocarditis is very diverse, we can find cases from patients not requiring treatment to patients with acute cardiac failure who may even require cardiac transplantation or without it, this will inevitably lead to death. In a systematic review conducted by Castiello et al., while 28 patients could have been discharged from hospital after their recovery, 5 of them died [1]. Despite the low specificity of elevated cardiac markers related to myocarditis, high levels of troponin and brain natriuretic peptide have been associated with a worse prognosis [23]. Given the inflammatory response to which COVID-19 infection leads, an increased risk of residual myocardial damage and sequelae not yet elucidated have been observed in survivors despite their recovery [4].

Of the 323 reported cases of confirmed postvaccination myocarditis or pericarditis (regarding the CDC definition), 96% required hospitalization, although most of them were discharged without complications [26]. In children who develop myocarditis in the context of multisystem inflammatory syndrome, a higher frequency of intensive care admissions has been observed. In these cases, there were factors described as well as lymphopenia or plaquetopenia, high levels of troponin, ferritin, C-reactive protein, brain natriuretic peptide, and N-terminal pro BNP type B, D-dimer or interleukin-6 [16].

## 9. Prognosis

It is a fact that although interest revolves around inflammation and cardiac damage, the long-term effects after recovery from myocarditis remain unclear. [23]. The mechanism of myocardial dysfunction is still not well known, as it may be secondary to myocarditis itself or to the systemic inflammatory state caused by cytokine release or thrombotic microvascular angiopathy. In any case, it is always recommended for ischemia to be ruled, and the myocardial dysfunction per se carries a worse prognosis [34]. It should be clarified that while the myocardial involvement in COVID-19 infection has been associated with a worse prognosis, myocarditis as an isolated entity does not necessarily entail a poor prognosis [1]. Furthermore, in a recent study involving 1080 children, the relationship between the outcome of myocarditis and race was not found [16]. 

With regard to revaccination after myocarditis related to SARS-CoV-2 vaccination, while definitive evidence remains lacking, general recommendations still advocate against the administration of subsequent doses of any SARS-CoV-2 vaccine. In cases where, after a risk assessment, the decision is made to administer a subsequent SARS-CoV-2 vaccine dose, the recommendation is to wait until the episode of myocarditis/pericarditis has been resolved (i.e., resolution of symptoms and no evidence of ongoing heart inflammation). However, people who have a history of myocarditis or pericarditis unrelated to SARS-CoV-2 vaccination may receive the SARS-CoV-2 vaccine after the episode of myocarditis/pericarditis has been completely resolved [42].

## 10. Conclusions

In conclusion, a wide range of cardiovascular complications, including myocarditis and pericarditis, must be suspected in patients with cardiac symptoms after COVID-19 infection or SARS-CoV-2 vaccination. The diagnosis of myocarditis related to COVID-19 and SARS-CoV-2 vaccination is primarily clinical, including a thorough differential diagnosis. At present, EMB is not routinely recommended as a diagnostic protocol given its low sensitivity. The treatment is essentially supportive, in which the antiviral therapy and the immunomodulators could be indicated in the hyperinflammatory phase, with limited scientific evidence. When myocarditis is observed in the setting of the multisystem inflammatory syndrome, the outcome and evolution become drastically worse. Finally, vaccination against SARS-CoV-2 in all age groups and sexes, based on the benefit–risk assessment, is highly recommended. 

Despite scientific advances in the last two years, certain knowledge gaps remain. Further clinical research is needed in order to better understand the disease, to achieve accurate and early diagnosis, and to apply the necessary and specific treatment in each clinical situation. First of all, the authors highlight the relevance of translational research as the key tool for transferring molecular and histological knowledge into diagnostic instruments and therapeutic targets. In this way, a promising line of research is the evaluation of the role of TLR4 antagonists in myocarditis related to COVID-19. According to the high risk related to EMB, we encourage the major use of autopsies. The pathological information that autopsies offer cannot be dismissed, even the low prevalence of myocarditis findings in previous autopsies. Clinical research is essential for assessing the efficacy of new therapeutical approaches. Properly designed studies are needed, in which strict diagnostic criteria for myocarditis and the administration of treatments in a specific time frame allow us to obtain hard clinical evidence.

## Figures and Tables

**Table 1:** Total references selected and classified by the pandemic wave.

Type	Total References	First Wave	Second Wave	Third Wave	Fourth Wave	Fifth Wave	Sixth Wave
Case report	[6,7,8,9,10,11,12]	-	[12]	[11]	-	[6,7,8,9,10]	-
Free full text	[13,14,15]	-	-	[13]	-	[14]	[15]
Observational study	[5,16]	-	-	-	[16]	-	[5]
Clinical study	[17,18,19,20,21,22]		[20]	[19]	-	[17,18,21]	[22]
Systematic review	[1,2,3,4,23,24,25,26,27,28,29,30,31,32,33,34]	-	[24]	[2,3,29,30,31,32,34]	[4,23,33]	[26]	[1,25,27,28]
Meta-analysis	[35]	[35]	-	-	-	-	-
Multicenter study	[36,37,38,39]	-	[38]	-	-	[36]	[37,39]
Practice Guideline	[40,41]	[40,41]	-	-	-	-	-

The reporting period has been divided in turn into the following pandemic waves: first wave = until June of 2020; second wave = from July to December of 2020; third wave = from January to March of 2021; fourth wave = from April to June of 2021; fifth wave = from July to October of 2021; sixth wave = from November of 2021 to February of 2022.

**Table 2:** Description of myocarditis and pericarditis cases from articles included in this review.

Article	Inclusion Period	Myocarditis Related to COVID-19	Pericarditis Related to COVID-19	Myocarditis Related to SARS-CoV-2 Vaccination	Pericarditis Related to SARS-CoV-2 Vaccination
Castiello et al. [1]	December 2019 to September 2020	38 patients (12 patients confirmed by BEM, 25 patients confirmed by CMR)			
Oster et al [5]	December 2020 to August 2021			1626 patients (223 patients confirmed by CMR)	
Hajjo et al. [17]	From 1990 to September 2021			1579 patients	1063 patients
Bozkurt et al. [26]	From 1990 to June 2021				484 patients with myocarditis/pericarditis
Haussner et al. [27]	February 2020 to November 2020	51 patients (5 patients confirmed by BEM, 25 patients by CMR)			
Nygaard et al. [37]	May 2021 to September 2021				15 patients
Sawalha et al. [29]	December 2019 to June 2020		14 patients with myocarditis/pericarditis (7 patients confirmed by CMR)		
Halushka et al. [32]	January to September 2020	277 autopsied hearts. 5 patients with definitive myocarditis,			
Larson et al. [7]	-	8 patients (8 patients confirmed by CMR)			
Blagova et al. [21]	March 2020 to March 2021	15 patients (6 patients confirmed by BEM, 10 patients by CMR)			

EMB = endomyocardial biopsy; CMR = cardiac magnetic resonance.

## Data Availability

The data presented in this study are available on request from the corresponding authors.

## Appendix A

**Table A1:** The Dallas criteria for myocarditis.

**Definition:** An inflammatory infiltrate of the myocardium with necrosis and/or degeneration of adjacent myocytes not typical of the ischemic damage associated with coronary artery disease
**First biopsy** − Myocarditis with/without fibrosis − Borderline myocarditis (repeat biopsy may be indicated) − No myocarditis
**Subsequent biopsy** − Ongoing (persistent) myocarditis with or without fibrosis − Resolving (healing) myocarditis with or without fibrosis − Resolved (healed) myocarditis with or without fibrosis
